# Comparison of the Efficiency of BLUP and GBLUP in Genomic Prediction of Immune Traits in Chickens

**DOI:** 10.3390/ani10030419

**Published:** 2020-03-03

**Authors:** Jin Zhang, Jie Wang, Qinghe Li, Qiao Wang, Jie Wen, Guiping Zhao

**Affiliations:** 1 Institute of Animal Sciences, Chinese Academy of Agricultural Sciences, Beijing 100193, China; zhangjin0913@126.com (J.Z.); wangjie4007@126.com (J.W.); liqinghe@caas.cn (Q.L.); wangqiao01@cass.cn (Q.W.); wenjie@caas.cn (J.W.); 2 State Key Laboratory of Animal Nutrition, Beijing 100193, China

**Keywords:** immune traits, SRBC, H/L, chicken, BLUP, GBLUP, cross-validation

## Abstract

**Simple Summary:**

With the rapid development of the poultry industry, outbreaks of certain avian diseases such as avian influenza, Newcastle disease, and salmonellosis can cause severe loss to local economies. An effective method to avoid this problem may be developing resistant lines on the basis of immune response traits. In our study, when the number of genotyped reference animals was small, the traditional pedigree-based method (BLUP) was more efficient than the genetic linear unbiased prediction of genomes (GBLUP) method in predicting the genetic parameters of sheep red blood cell antibody titer (SRBC), the ratio of heterophils to lymphocytes (H/L), the spleen immune index (SII) and the spleen weight at 100 d (SW) in Beijing oil chickens.

**Abstract:**

Poultry diseases pose a large threat to poultry production. Selection to improve immune traits is a feasible way to prevent and control avian diseases. The objective of this study was to investigate the efficiency of estimation of genetic parameters for antibody response to avian influenza virus (Ab-AIV), antibody response to Newcastle disease virus (Ab-NDV), sheep red blood cell antibody titer (SRBC), the ratio of heterophils to lymphocytes (H/L), immunoglobulin G (IgG), the spleen immune index (SII), thymus immune index (TII), thymus weight at 100 d (TW) and the spleen weight at 100 d (SW) in Beijing oil chickens, by using the best linear unbiased prediction (BLUP) method and genomic best linear unbiased prediction (GBLUP) method. The phenotypic data used in the two methods were the same and were from 519 individuals. With the BLUP model, Ab-AIV, Ab-NDV, SRBC, H/L, IgG, TII, and TW had low heritability ranging from 0.000 to 0.281, whereas SII and SW had high heritability of 0.631 and 0.573. With the GBLUP model, all individuals were genotyped with Illumina 60K SNP chips, and Ab-AIV, Ab-NDV, SRBC, H/L and IgG had low heritability ranging from 0.000 to 0.266, whereas SII, TII, TW and SW had moderate heritability ranging from 0.300 to 0.472. We compared the prediction accuracy obtained from BLUP and GBLUP through 50 time 5-fold cross-validation (CV), and the results indicated that BLUP provided a slightly higher accuracy of prediction than GBLUP in this population.

## 1. Introduction

In poultry breeding, the main objective traits are growth and reproduction traits, whereas, little attention has been paid to the selection of immune traits. Regular immunization is a large expense in poultry production, and the problem of poultry diseases remains very serious [1,2,3]. Selection of immune traits is able to not only improve poultry disease resistance but also effectively control production costs. In chicken breeding, immune performance evaluation commonly includes the following: antibody response to avian influenza virus (Ab-AIV), antibody response to Newcastle disease virus (Ab-NDV), sheep red blood cells (SRBC), ratio of heterophils to lymphocytes (H/L), immune organ index (spleen immune index (SII), thymus immune index (TII), thymus weight at 100 d (TW) and spleen weight at 100 d (SW)) and serum immunoglobulin G (IgG).

SRBC is a non-pathogenic multivalent antigen that has been widely used in animal humoral immune response studies, it reflects the level of antibodies produced by the body [4]. SRBC antibodies are positively correlated with infection with Newcastle disease virus, Staphylococcus aureus and so forth [5,6]. Several studies have shown that the selection of this trait can improve the genetic resistance of poultry [7,8]. H/L is an immune trait that directly responds to the corresponding immune effects at the cellular level. Individuals with low H/L values usually have high resistance to heat stress [9,10,11]. Al-Murrani [10] has used this value as a selection criterion for *Salmonella* typhimurium resistance and has suggested that H/L is highly heritable in chickens. In general, SRBC and H/L can be more suitable as indicators for the breeding of new strains with high disease resistance. Both can measure the body’s general disease resistance and have stable heritability; consequently, selecting the traits can improve general disease resistance in chickens.

Accurate and reliable estimation of genetic parameters is a prerequisite for rational breeding programs during the animal breeding process. In recent years, with the detection of single nucleotide polymorphism (SNP) markers and the rapid development of biochip technology, new genetic-prediction methods have been developed, notably the genetic linear unbiased prediction of genomes (GBLUP) method [12]. Genomic selection represents a revolution beyond traditional breeding and is based on calculation of the genomic estimated breeding value (GEBV) according to genome-wide marker information [13]. Generally, genomic selection achieves a higher estimation accuracy than the conventional estimated breeding value based on pedigree [14,15,16]. This selection method is useful for traits that are difficult and expensive to measure [17,18], such as the avian influenza antibody titer, the immune response against sheep red blood cells and the Newcastle disease antibody titer; it allows for higher accuracy of the estimated breeding value to be obtained. Several reports have demonstrated that genomic selection is more advantageous than the conventional method on the basis of pedigree information in poultry [19,20,21].

The objective of this study was to estimate genetic parameters of nine immune traits and to compare the accuracy of BLUP and GBLUP in genomic prediction on immune traits in Beijing oil chickens.

## 2. Materials and Methods

### 2.1. Ethics Statement

Ethical approval on animal care and experimental procedures were performed in accordance with the Animal Ethics Committee of the Institute of Animal Sciences, Chinese Academy of Agricultural Sciences (IAS-CAAS, Beijing, China) with the following reference number: IASCAASAE-03.

### 2.2. Population and Phenotypes

The data were collected from 519 Beijing oil chicken cocks raised at the Chang ping Experimental Base of Institute of Animal Sciences. The Beijing oil chicken is an indigenous Chinese breed characterized by slow growth and high meat quality. All birds were from the same batch, the offspring of 53 cocks and 199 hens. At 100 d of age, the chickens were killed, and blood and serum samples were collected. We recorded immune traits including Ab-AIV, Ab-NDV, SRBC, H/L, IgG, SII, TII, TW and SW (Table 1). 

Ab-AIV, AI-NDV, and SRBC antibody levels in the serum samples were measured with hemagglutination inhibition tests according to previous studies [22,23,24,25] and were expressed as the log_2_ value of the corresponding dilutions. H/L was measured by using blood smears made with fresh blood [24]. Briefly, after staining with Wright’s Giemsa solution, the heterophils and lymphocytes on the blood smears were counted under a light microscope. IgG in the serum samples was measured with an indirect ELISA kit (Cusabio, Wuhan, China) [23]. The weights of the spleen, thymus and carcass were measured to detect the SII, TII, TW and SW.

SII (%) = weight of spleen(g)/weight of carcass(g)

TII (%) = weight of thymus(g)/weight of carcass(g)

### 2.3. Genotype Data

Genomic DNA was extracted from blood samples with a conventional phenol method, and then genotyping was performed with 60K SNP chips (Illumina, San Diego, CA, USA). All 519 chickens with immune trait records were genotyped. PLINK (V1.07) software was used to control for the quality of chip genotype data, with individual call rates > 90%, SNP locus call rates > 95% and minor allele frequency > 1%. After quality control, 46 876 SNP markers and 519 individuals were used.

### 2.4. Statistical Model

In this study, two methods, a pedigree-based BLUP method with a pedigree-based relationship matrix and a GBLUP method based on genomic relationship matrix, were used to predict the breeding value.

#### 2.4.1. BLUP

The traditional animal model with a pedigree-based relationship matrix was applied to predict breeding values for each trait separately [26]. The model was defined as:

(1) y=1μ+Za+e

where **y** was the vector of phenotypic values (Ab-AIV, Ab-NDV, SRBC, H/L, IgG, SII, TII, TW and SW), μ was the overall mean, **1** was a vector of 1, **a** was the vector of additive genetic effects, following a normal distribution of N(0, Aσ_a_^2^), where A was the pedigree-based genetic relationship matrix, and σ_a_^2^ was the variance of additive genetic effects. **Z** was the incidence matrix associating **a** with **y**, and **e** was the vector of random residuals with distribution of N (0, Iσ_e_^2^), in which I was the identity matrix, and σ_e_^2^ was the variance of residuals.

#### 2.4.2. GBLUP

The GBLUP model was used to predict the GEBV of all genotyped individuals [12].

(2) y=1μ+Zg+e

where the definitions of **y**, **Z** and **e** were the same as those in the BLUP model, **g** was the vector of genomic breeding values to be estimated, following a normal distribution of N(0, Gσ_g_^2^), in which σ_g_^2^ was the variance of additive genetic effects, and G was the marker-based genomic relationship matrix. **Z** was the incidence matrix linking **g** to **y**, and **e** was the vector of random residuals following a normal distribution of N (0, Iσ_e_^2^), in which σ_e_^2^ was the variance of residuals. The estimation of variance components and the prediction of breeding values were performed with the Asreml package [27].

### 2.5. Cross-Validation

Cross-validation (CV) is usually used to obtain a reliable and stable model, and to evaluate the quality of the model [28,29]. In this study, 5-fold CV was applied to assess the accuracy of the genomic predictions. The method to create subsets of sampling was used according to a previous study [30]. Briefly, 519 birds were randomly divided into five subsets. In each fold of validation, one data set was used as the test data set, and the other four data sets were used as reference datasets. All data sets were used to build a model. To account for sampling variation, splitting was repeated 50 times. The CV was performed with the Asreml package and caret package [27,31].

Predictive Ability: Correlation between phenotype and breeding value (y: phenotype; $\bar{y}$: breeding value)

(3) $$Corr\left(y,\;\bar{y}\right)$$

Prediction Accuracy: Accuracy of breeding values (g: actual breeding value; $\bar{g}$: predicted breeding values; h: Arithmetic square root of heritability

(4) $$Corr\left(g,\;\bar{g}\right)=\frac{Corr\left(y,\;\bar{y}\right)}{h}$$

## 3. Results

### 3.1. Descriptive Statistics and Estimates of Genetic Parameters

**Figure 1:**
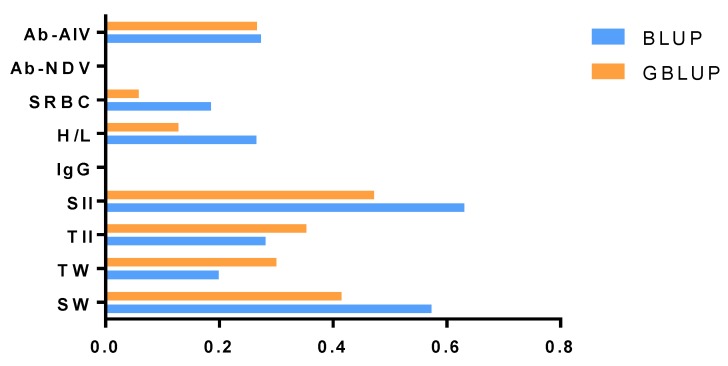
Comparison of heritability estimated with the best linear unbiased prediction (BLUP) model (Blue bar) and genetic linear unbiased prediction of genomes (GBLUP) model (orange bar). The *x*-axis is the value of heritability, and the *y*-axis is the name of the immune trait.

The estimated genetic parameters for the nine immune traits were presented in Table 2 and Table 3. According to the heritability of the nine immune traits, we compared the differences between the heritability estimated with the BLUP and GBLUP methods in Figure 1. The estimates of heritability of SRBC, H/L, SII and SW were generally higher with the BLUP model than the GBLUP model.

Using the entire dataset, we found that the heritability of the nine immune traits ranged from 0 to 0.631 with the pedigree-based BLUP model. Across the nine analyzed traits, the estimated heritability of SII was the highest, at 0.631. The standard error (SE) values of estimated heritability for all traits were less than 0.110. The heritability of Ab-NDV and IgG was almost 0, and Ab-AIV, SRBC, H/L, TII and TW had low heritability ranging from 0.185 to 0.281. However, SII and SW had high heritability of 0.631 and 0.573, respectively. With the GBLUP model, SRBC, Ab-NDV and IgG had the lowest heritability. Ab-AIV and H/L had low heritability of 0.266 and 0.128, respectively. SII, TII, TW and SW had moderate heritability ranging from 0.300 to 0.472. The SE of estimated heritability was less than 0.090.

### 3.2. The Accuracy of Genomic Prediction with the BLUP and GBLUP Methods

As shown in Table 4, the accuracy of predictions from 50 time 5-fold CV was calculated on SRBC and H/L immune traits through a pedigree-based BLUP model and GBLUP model. In the two scenarios, the GBLUP model had a lower predictive ability. The prediction accuracy between the conventional model and genomic model differed slightly, for SRBC, the mean of prediction accuracy from the BLUP was 0.091 higher than GBLUP.

### 3.3. Breeding Values of Various Traits Predicted with BLUP and GBLUP for Birds with Low or High SRBC and H/L 

Table 5 and Table 6 show the means for different phenotypic traits on the basis of 30 efficient and 30 inefficient values of SRBC and H/L. These individuals with extreme values were selected according to estimated breeding values from BLUP or GBLUP models. For SRBC, as shown in Table 5, comparison of phenotypic values for the 30 birds with extreme values indicated that the nine immune traits had no significant differences, but there was a slight difference between the two methods, and the mean phenotypic value of SRBC with BLUP was higher than that with GBLUP (*p* = 0.081), For H/L, as shown in Table 6, there was also no significant difference between the two methods, but the phenotypic mean of H/L for the 30 birds with efficient values was 0.134 with BLUP but 0.153 with GBLUP. Thus, selecting for SRBC and H/L was more efficient with the BLUP than the GBLUP model.

## 4. Discussion

In this study, the estimated heritability of nine immune traits ranged from 0.00 to 0.631 with a pedigree-based BLUP model and from 0.000 to 0.472 with the GBLUP model. Therefore, the estimate of heritability was higher with the BLUP method. For the BLUP model, the estimates of heritability for the two main selected immune traits, SRBC and H/L, were 0.185 and 0.265, respectively. SRBC and H/L had low heritability in Beijing oil chickens, which is in line with findings from previous studies. Boa-Amponsem has reported that maternal antibody levels affect antibody titers in next-generation chickens, and the heritability of chickens responding to antibodies produced by SRBC is relatively stable [32]. Al-Murrani has demonstrated that H/L is highly heritable in chickens [33]. The heritability of Ab-NDV and Ab-AIV is 0.478 and 0.301 according to a report of Liu, et al. [30], but the estimates of heritability of the two traits in our study were lower, at 0 and 0.273, respectively, a finding possibly associated with the genetic background of the chickens. In the spleen and thymus, which are important immune organs in birds, the estimated heritability of the immune organ index was 0.631 and 0.281, the estimated heritability of immune organ weight was 0.573 and 0.199, respectively, thus indicating that the two immune traits are positively correlated with spleen weight and thymus weight at 100 d. With the GBLUP model, the estimated heritability of nine immune traits was lower than that with the BLUP model.

The SEs of heritability estimates were low. Although the dataset in our study was small, there are some possible reasons that may explain this finding. First, the individuals were from a single farm and lived in the same environment during the same period; thus, the model was simple, and relatively few parameters needed to be estimated. Second, the dataset had good structure in estimating genetic parameters because of the appropriate numbers of half-siblings and full-siblings.

We selected SRBC and H/L as the target immune traits to detect the accuracy through 50 time 5-fold CV. Compared with the GBLUP method, the traditional BLUP method had slightly higher accuracy for the two immune traits, and the accuracy of prediction for H/L was higher than that for SRBC. Our results differed from those in previous reports [34,35], in which genomic information has been found to outperform pedigree in estimating relatedness for two possible reasons. On the one hand, the heritability for H/L and SRBC traits was below moderate, thus enabling sufficient accuracy of the traditional BLUP method, and the improvement from genomic prediction was not as large as expected. On the other hand, owing to the existence of missing heritability, the 60K SNP chip cannot be used to accurately determine the genetic relationships between individuals [36]. Moreover, one study has indicated that the expected value of BLUP might be high [37]. Another study has also indicated that prediction accuracy increases monotonically with the size of the training population [38,39], thus also suggesting a reason for the slightly lower accuracy of GBLUP than BLUP in this study (519 individuals).

## 5. Conclusions

Our research aimed to analyze the efficiency of BLUP and GBLUP in genomic prediction on immune traits in chickens. Estimates of heritability of SRBC, H/L, SII and SW were higher with the BLUP model than the GBLUP model. Importantly, the BLUP model led to slightly higher accuracy in genetic parameter prediction than the GBLUP model in our study.

## Figures and Tables

**Table 1:** Descriptive statistics of nine immune traits.

Trait ^1^	N-Obs ^2^	Average	SD ^3^	Min Value	Max Value
Ab-AIV	455	9.479	1.558	5.000	12.000
Ab-NDV	384	5.505	1.262	3.000	9.000
SRBC	519	7.788	1.191	4.000	10.000
H/L	519	0.438	0.294	0.024	1.579
IgG	466	542.855	219.233	144.670	1754.730
SII	519	0.150	0.037	0.059	0.303
TII	514	0.477	0.174	0.043	1.045
TW	515	7.217	2.786	0.460	17.610
SW	519	2.269	0.622	0.800	4.360

^1^ Ab-AIV, antibody response to avian influenza virus; Ab-NDV, antibody response to Newcastle disease virus; SRBC, sheep red blood cell antibody titer; H/L, the ratio of heterophils to lymphocytes; IgG (ng/ml), immunoglobulin G; SII (%), spleen immune index; TII (%), thymus immune index; TW (g), thymus weight at 100 d; SW (g), spleen weight at 100 d.; ^2^ number of observations.^3^ Standard deviation.

**Table 2:** Genetic parameters estimated from all data with the best linear unbiased prediction (BLUP) model.

Trait ^1^	σ_a_^2^		σ_e_^3^		h ^4^	
	Component	Std. Error	Component	Std. Error	Estimate	Std. Error
Ab-AIV	0.662	0.261	1.764	0.245	0.273	0.101
Ab-NDV	0	0	1.593	0.115	0	0
SRBC	0.263	0.128	1.157	0.131	0.185	0.087
H/L	0.022	0.009	0.064	0.008	0.265	0.100
IgG	0.006	0	9409.678	617.111	0	0
SII	0.001	0	0.001	0	0.631	0.110
TII	0.009	0.003	0.022	0.003	0.281	0.098
TW	1.546	0.722	6.233	0.726	0.199	0.089
SW	0.223	0.052	0.166	0.038	0.573	0.109

^1^ Ab-AIV, antibody response to avian influenza virus; Ab-NDV, antibody response to Newcastle disease virus; SRBC, sheep red blood cell antibody titer; H/L, the ratio of heterophils to lymphocytes; IgG (ng/ml), immunoglobulin G; SII (%), spleen immune index; TII (%), thymus immune index; TW (g), thymus weight at 100 d; SW (g), spleen weight at 100 d. ^2^ the variance of additive genetic effects. ^3^ the variance of residuals. ^4^ heritability.

**Table 3:** Genetic parameters estimated from all data with the genetic linear unbiased prediction of genomes (GBLUP) model.

Trait ^1^	σ_g_^2^		σ_e_^3^		h ^4^	
	Component	Std. Error	Component	Std. Error	Estimate	Std. Error
Ab-AIV	0.644	0.224	1.774	0.210	0.266	0.086
Ab-NDV	0	0	1.593	0.115	0	0
SRBC	0.082	0.087	1.337	0.116	0.058	0.061
H/L	0.011	0.006	0.075	0.007	0.128	0.065
IgG	0.003	0	9409.681	617.111	0	0
SII	0.001	0	0.001	0	0.472	0.083
TII	0.011	0.003	0.020	0.002	0.353	0.086
TW	2.332	0.701	5.434	0.619	0.300	0.082
SW	0.158	0.036	0.223	0.028	0.415	0.080

^1^ Ab-AIV, antibody response to avian influenza virus; Ab-NDV, antibody response to Newcastle disease virus; SRBC, sheep red blood cell antibody titer; H/L, the ratio of heterophils to lymphocytes; IgG (ng/ml), immunoglobulin G; SII (%), spleen immune index; TII (%), thymus immune index; TW (g), thymus weight at 100 d; SW (g), spleen weight at 100 d. ^2^ the variance of additive genetic effects. ^3^ the variance of residuals. ^4^ heritability.

**Table 4:** The accuracy of prediction for sheep red blood cells (SRBC) and heterophils to lymphocytes (H/L) traits in 50 time 5-fold cross-validation (CV).

Trait ^1^	SRBC			H/L		
	Predictive Ability	Prediction Accuracy	Unbiasedness	Predictive Ability	Prediction Accuracy	Unbiasedness
BLUP	0.132 ± 0.0242 ^2^	0.307 ± 0.056	1.000 ± 0.001	0.185 ± 0.030	0.326 ± 0.528	0.999 ± 0.006
GBLUP	0.052 ± 0.027	0.216 ± 0.112	1.000 ± 0.000	0.119 ± 0.025	0.333 ± 0.069	0.999 ± 0.004
*P*-value ^3^	1.082 × 10 ^−28^	1.082 × 10 ^−28^	9.282 × 10 ^−01^	2.309 × 10 ^−20^	2.309 × 10 ^−20^	9.164 × 10 ^−01^

^1^ SRBC, sheep red blood cell antibody titer; H/L, the ratio of heterophils to lymphocytes; ^2^ mean ± SE; ^3^ Student’s t-test.

**Table 5:** Mean and standard deviation of breeding values of various traits predicted from the two methods for birds with low or high SRBC.

Model	Group ^1^	Ab-AIV ^2^	SRBC	Ab-NDV	H/L	IgG	SII	TII	TW	SW
BLUP	SRBCeff	9.240 ± 1.964	9.567 ± 0.504	5.500 ± 1.303	0.446 ± 0.358	651.166 ± 330.626	0.140 ± 0.036	0.438 ± 0.152	6.541 ± 2.554	2.054 ± 0.585
GBLUP	SRBCeff	9.346 ± 1.788	9.300 ± 0.651	5.500 ± 1.251	0.442 ± 0.358	545.036 ± 252.567	0.141 ± 0.029	0.450 ± 0.163	6.794 ± 0.714	2.103 ± 0.517
	*p*-value ^3^	0.841	0.081	1.000	0.968	0.191	0.898	0.780	0.714	0.734
BLUP	SRBCineff	9.708 ± 1.301	5.533 ± 0.860	5.818 ± 1.468	0.611 ± 0.352	558.716 ± 296.872	0.151 ± 0.049	0.440 ± 0.117	6.757 ± 2.012	2.297 ± 0.793
GBLUP	SRBCineff	9.870 ± 1.180	5.933 ± 1.048	5.789 ± 1.548	0.512 ± 0.240	591.997 ± 282.724	0.146 ± 0.047	0.466 ± 0.141	7.078 ± 2.287	2.201 ± 0.780
	*p*-value	0.659	0.112	0.952	0.208	0.693	0.678	0.440	0.573	0.639

^1^ SRBCeff, the top 30 chickens with efficient values and high anti-SRBC antibody titer; SRBCineff, the bottom 30 chickens with inefficient values and low anti-SRBC antibody titer; ^2^ Ab-AIV, antibody response to avian influenza virus; SRBC, sheep red blood cell antibody titer; Ab-NDV, antibody response to Newcastle disease virus; H/L, the ratio of heterophils to lymphocytes; IgG, immunoglobulin G (ng/ml); SII (%), spleen immune index; TII (%), thymus immune index; TW (g), thymus weight at 100 d; SW (g), spleen weight at 100 d; ^3^ Student’s t-test.

**Table 6:** Mean and standard deviation of breeding values of various traits predicted from the two methods for birds with low or high H/L.

Model	Group ^1^	Ab-AIV ^2^	SRBC	Ab-NDV	H/L	IgG	SII	TII	TW	SW
BLUP	H/Leff	9.393 ± 1.792	7.600 ± 1.102	5.304 ± 1.185	0.134 ± 0.062	537.249 ± 207.421	0.145 ± 0.037	0.528 ± 0.142	7.614 ± 2.077	2.114 ± 0.582
GBLUP	H/Leff	9.500 ± 1.679	7.733 ± 1.112	5.455 ± 1.101	0.153 ± 0.075	544.312 ± 226.187	0.137 ± 0.036	0.480 ± 0.181	6.904 ± 2.701	1.967 ± 0.618
	*p*-value ^3^	0.822	0.643	0.662	0.296	0.905	0.408	0.266	0.264	0.347
BLUP	H/Lineff	9.818 ± 1.220	7.567 ± 1.194	5.750 ± 1.446	1.100 ± 0.340	585.091 ± 181.843	0.165 ± 0.046	0.479 ± 0.172	7.595 ± 2.954	2.616 ± 0.797
GBLUP	H/Lineff	9.348 ± 1.613	7.367 ± 1.129	5.286 ± 1.309	0.949 ± 0.393	580.645 ± 238.276	0.167 ± 0.046	0.473 ± 0.195	7.518 ± 3.564	2.634 ± 0.856
	*p*-value	0.278	0.508	0.287	0.115	0.942	0.873	0.903	0.928	0.932

^1^ H/Leff, the top 30 chickens with efficient values and low ratio of heterophils to lymphocytes; H/Lineff, the bottom 30 chickens with inefficient values and low ratio of heterophils to lymphocytes; ^2^ Ab-AIV, antibody response to avian influenza virus; SRBC, sheep red blood cell antibody titer; Ab-NDV, antibody response to Newcastle disease virus; H/L, the ratio of heterophils to lymphocytes; IgG, immunoglobulin G (ng/ml); SII (%), spleen immune index; TII (%), thymus immune index; TW (g), thymus weight at 100 d; SW (g), spleen weight at 100 d; ^3^ Student’s t-test.
